# Generation and protective efficacy of a cold-adapted attenuated avian H9N2 influenza vaccine

**DOI:** 10.1038/srep30382

**Published:** 2016-07-26

**Authors:** Yandi Wei, Lu Qi, Huijie Gao, Honglei Sun, Juan Pu, Yipeng Sun, Jinhua Liu

**Affiliations:** 1 Key Laboratory of Animal Epidemiology and Zoonosis, Ministry of Agriculture, College of Veterinary Medicine, and State Key Laboratory of Agrobiotechnology, China Agricultural University, Beijing, 100193, China

## Abstract

To prevent H9N2 avian influenza virus infection in chickens, a long-term vaccination program using inactivated vaccines has been implemented in China. However, the protective efficacy of inactivated vaccines against antigenic drift variants is limited, and H9N2 influenza virus continues to circulate in vaccinated chicken flocks in China. Therefore, developing a cross-reactive vaccine to control the impact of H9N2 influenza in the poultry industry remains a high priority. In the present study, we developed a live cold-adapted H9N2 influenza vaccine candidate (SD/01/10-ca) by serial passages in embryonated eggs at successively lower temperatures. A total of 13 amino acid mutations occurred during the cold-adaptation of this H9N2 virus. The candidate was safe in chickens and induced robust hemagglutination-inhibition antibody responses and influenza virus–specific CD4^+^ and CD8^+^ T cell immune responses in chickens immunized intranasally. Importantly, the candidate could confer protection of chickens from homologous and heterogenous H9N2 viruses. These results demonstrated that the cold-adapted attenuated H9N2 virus would be selected as a vaccine to control the infection of prevalent H9N2 influenza viruses in chickens.

The H9N2 avian influenza virus (AIV) was first identified in chicken farms in Guangdong Province of China in 1992^1^. Since then, H9N2 viruses have spread to the whole country and become the most prevalent subtype of influenza virus in chickens in China, resulting in great economic losses due to reduced egg production or high mortality associated with co-infection with other pathogens^2^,^3^,^4^,^5^,^6^. H9N2 virus in avian species has been repeatedly transmitted to mammals and humans, resulting increasing public threats^7^. H9N2 viruses also serve as vehicles by donating their gene segments to other emerging influenza A viruses, including H5N2^8^, H6N1^9^, H7N7^8^, H7N9^10^,^11^,^12^, and H10N8^13^,^14^ viruses. Among these emerging viruses, H7N9 subtype viruses resulted in 722 human infections and 286 deaths as of 25 February 2016 (http://www.who.int/influenza/human_animal_interface/HAI_Risk_Assessment/en/) and caused a disaster to poultry industry in China. Thus, developing methods to control the circulation of H9N2 viruses should be given priority.

To prevent H9N2 avian influenza virus infection in chickens, a vaccination program using inactivated oil-emulsion vaccines has been ongoing in China since 1998^4^. However, H9N2 outbreaks have continued to occur over the past two decades^15^,^16^. At least four different antigenic groups have been identified among H9N2 viruses in chickens in China, resulting in failure of immunization by inactivated vaccines^4^,^16^,^17^,^18^. Moreover, manufacturers instructed farmers to conduct the first dose of immunization on 1-week-old chickens with oil-emulsion inactivated H9N2 vaccines; however, these types of vaccines require 20 days to be effective^19^. H9N2 influenza frequently occurs in 20–30 day old chickens that lack maternally transferred antibodies or inactivated vaccine induced protection^20^. Therefore, it is difficult to use inactivated oil-emulsion vaccines to prevent H9N2 influenza in chickens. Thus, developing live attenuated vaccines conferring protection against antigenic drift variants would be a better choice to control H9N2 influenza in poultry, in China.

When compared with inactivated vaccines, live attenuated influenza vaccines (LAIVs) can elicit a broader range of virus-specific immune responses, including mucosal, serum antibody and cell-mediated responses, increasing the likelihood of generating broadly cross-reactive responses that may be effective against multiple virus strains^21^. In the United States, live attenuated reassortant vaccines have been approved for vaccination of humans to control human H1N1 and H3N2 influenza A viruses and influenza B viruses^22^. Live attenuated H2N2, H3N8, H5N1, H7N7, and H7N9 influenza virus candidate vaccines have been shown to be safe and effective at conferring protection against wild-type viruses in mice and ferrets^23^,^24^,^25^,^26^,^27^,^28^. In Korea, a cold-adapted attenuated H9N2 A/chicken/Korea/S1/03 vaccine strain was developed and experimentally shown to protect against wild type virus challenge^29^. H9N2 viruses circulating in Korea belong to the A/duck/Hong Kong/Y439/1997-like group, while H9N2 viruses circulating in chickens in China belong to the A/chicken/Hong Kong/Y280/97-like group^2^,^30^,^31^,^32^. H9N2 viruses isolated in Korea are phylogenetically and antigenically distinct from those viruses circulating in China^32^. Thus, it is necessary to develop a LAIV derived from the prevailing Chinese H9N2 virus.

In this study, we obtained a cold-adapted attenuated H9N2 influenza vaccine candidate by gradually lowering temperatures to 25 °C in eggs. The humoral and cellular immune responses induced by the cold-adapted H9N2 virus were analyzed. Furthermore, protective efficacy of the cold-adapted virus against wild H9N2 influenza viruses belonging to different HA lineages circulating in China was evaluated.

## Results

### Generation and phenotypes of cold-adapted H9N2 vaccine strain

**Figure 1:**
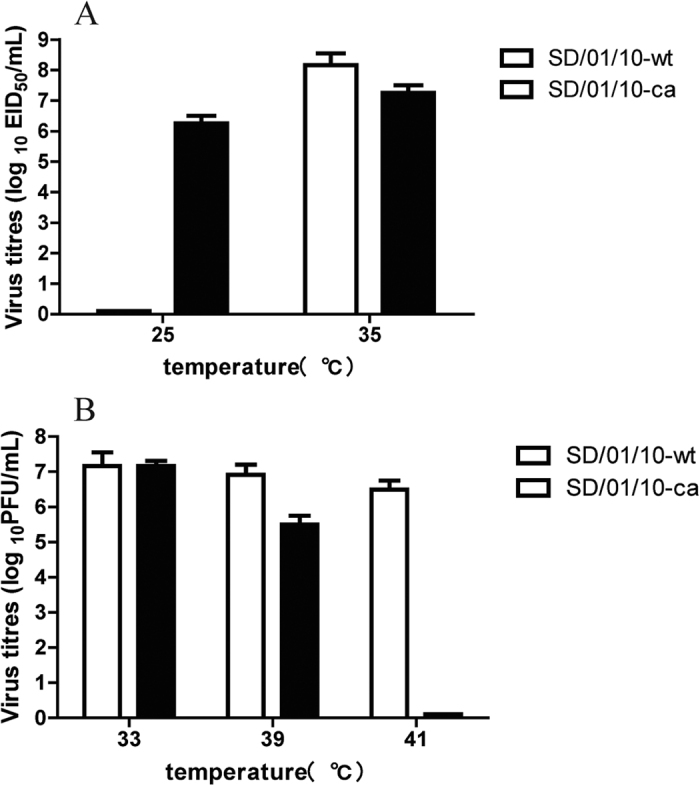
Cold adaptation and temperature sensitivity of cold-adapted H9N2 influenza virus. (**A**) Cold adaptation. Four HAU of cold-adapted (SD/01/10-ca) or wild-type H9N2 (SD/01/10-wt) influenza viruses were inoculated into 10-day-old SPF eggs (three SPF eggs/temperature), and the inoculated eggs were incubated at 25 °C and 35 °C for 48 h. The viral titers in the allantoic fluids were determined by log_10_ EID_50_/mL. Data are the mean virus titer of three independent experiments ± the standard errors. (**B**) Temperature sensitivity. The temperature sensitive phenotype of the SD/01/10-ca or SD/01/10-wt virus was determined by plaque assay in MDCK cells at 33 °C, 39 °C or 41 °C. Data are the mean virus titer of three independent experiments ± the standard errors.

The H9N2 cold-adapted SD/01/10-ca was generated by serial passages of SD/01/10-wt in SPF embryonated eggs at successively lower temperatures from 33 °C to 25 °C, then purified by limiting dilution in SPF eggs at 25 °C 10 times. To determine whether cold-adapted H9N2 vaccine candidate exhibited the *ca* phenotype, SD/01/10-ca or SD/01/10-wt was inoculated in SPF embryonated chicken eggs at 25 °C or 35 °C for 48 h, after which the virus titers were measured. As shown in Fig. 1A, both SD/01/10-ca and SD/01/10-wt grew to 7.0 log_10_ EID_50_/mL in eggs at 35 °C. The titer of SD/01/10-ca at 25 °C was 6.3 ± 0.3 log_10_ EID_50_/mL, while SD/01/10-wt was not able to grow at this temperature. To determine the *ts* phenotype, titers of SD/01/10-ca or SD/01/10-wt were assayed in MDCK cells at 33 °C, 39 °C or 41 °C. The SD/01/10-ca grew to 7.2 ± 0.1 and 5.5 ± 0.3 log_10_ PFU/mL at 33 °C and 39 °C, respectively, but failed to replicate at 41 °C. In contrast, SD/01/10-wt replicated well at 33 °C, 39 °C and 41 °C and reached to titers of 7.2 ± 0.4, 6.9 ± 0.3 and 6.5 ± 0.3 log_10_ PFU/mL, respectively (Fig. 1B). These results indicated that the SD/01/10-ca exhibited the *ca* and *ts* phenotype.

### Comparison of amino acid sequences between wild-type and cold-adapted H9N2 influenza viruses

To identify the mutation that occurred during cold adaptation of the H9N2 influenza virus, the genomes of the wild-type and cold-adapted viruses were sequenced and compared. A total of 13 mutations were detected in the cold adaptation of SD/01/10-wt; specifically PB2 (R136Q, G290C, R505L and P654S), PB1 (N292K, S361N and E739K), PA (D27N and E319K), HA (A150D and L216F), and NP (G17R), NS1 (G184R) (Table 1 and Fig. S1). A nonsense nucleotide mutation, T to C at position 853 in the M gene, was also included in this process. None of these mutations were observed in the previous cold-adapted viruses, including A/Leningrad/134/17/57 (H2N2), A/Leningrad/134/47/57 (H2N2), A/Ann Arbor/6/60 (H2N2) and A/chicken/Korea/S1/03 (H9N2)^29^,^33^,^34^, indicating that there were novel mutations responsible for cold adaptation of the influenza virus in the present study that were not recognized previously.

### Pathotype, replication and transmission of cold-adapted H9N2 influenza virus in chickens

To evaluate the virulence of SD/01/10-ca in chickens, SPF chickens were inoculated intranasally with 10^6^ EID_50_ of SD/01/10-ca and SD/01/10-wt virus. All chickens inoculated with SD/01/10-ca remained healthy, while chickens infected with SD/01/10-wt showed mild depression, anorexia, and diarrhea after 2 days post inoculation. SD/01/10-ca was only detected in the tracheas of inoculated chickens on 2 days post inoculation with titers of 1.8 ± 0.4 log_10_ EID_50_/mL, and was not detected in lungs (Table 2). In contrast, SD/01/10-wt replicated in both tracheas and lungs on 2, 3, 5, and 7 days post inoculation with peak titers of 4.4 ± 0.4 and 5.3 ± 0.9 log_10_ EID_50_/mL, respectively. These results indicated that the cold-adapted H9N2 vaccine strain exhibited attenuated pathotype in chickens.

To exclude the possibility that the cold-adapted H9N2 influenza virus can spread from vaccinated chickens to unvaccinated chickens, we evaluated its transmissibility in chickens. No viruses were detected in either oropharynx or cloacae of chickens that contacted the SD/01/10-ca infected chickens, while chickens that contacted SD/01/10-wt infected chickens shed viruses from both the oropharynx and cloacae on 2 and 4 days post inoculation (Table 2). These data suggested that the SD/01/10-ca may not be transmitted from the vaccinated chickens to naive chickens.

### Humoral and cellular immune responses to cold-adapted H9N2 virus in chickens

**Figure 2:**
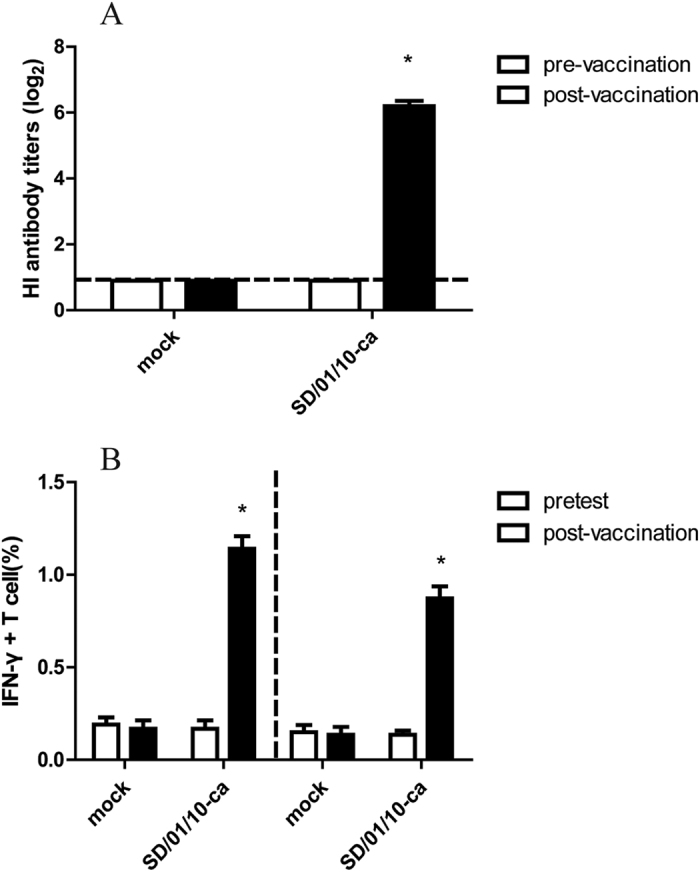
Immune responses induced by cold-adapted H9N2 vaccine in chickens. (**A**) SD-01-10-wt specific antibody levels assessed by HI assay. Groups of three-week-old SPF white Leghorn chickens (n = 36) were immunized intranasally with 10^6^ EID_50_ of cold-adapted H9N2 viruses in 0.2 mL or phosphate-buffered saline (mock immunized) in 0.2 mL. Serum HI antibodies were measured against SD-01-10-wt pre-vaccination and on day 14 after vaccination. Black dashed lines indicate the lower limit of detection. *P < 0.05, by one-way analysis of variance to evaluate the difference between the value tested pre vaccination and 14 days post vaccination. (**B**) The frequency of IFN-γ^+^CD4^+^ or IFN-γ^+^CD8^+^ T cells in the lungs after cold-adapted H9N2 virus vaccination. Groups of three-week-old SPF white Leghorn chickens (n = 5) were immunized intranasally with 0.2 mL 10^6^ EID_50_ of cold-adapted H9N2 or phosphate-buffered saline (mock immunized). The lungs of five SD-01-10-ca vaccinated and five mock-vaccinated chickens were harvested pre-vaccination and on day 7 post vaccination. IFN-γ^+^CD4^+^ or IFN-γ^+^CD8^+^ T cells after stimulation of the lung cells with SD-01-10-wt were measured by intracellular cytokine staining. Percentages of IFN-γ^+^CD4^+^ or IFN-γ^+^CD8^+^ T cells within CD4^+^ or CD8^+^ T cells were analyzed. *P < 0.05, by one-way analysis of variance of the difference between the value tested pre vaccination and 14 days post vaccination.

To assess the immunogenicity of SD/01/10-ca virus in chickens, serum were collected before vaccination and 2 weeks after delivery, while lungs were collected before vaccination and on day 7 post-vaccination and stimulated with SD/01/10-wt *in vivo* to determine the levels of virus specific IFN-γ–secreting T cells. As shown in Fig. 2A, each of the vaccinated chickens had robust HI antibody response against SD/01/10-wt at 2 weeks post-vaccination, with a mean titer of 2^6.2^, compared with the baseline levels before vaccination. Conversely, titers in mock-vaccinated animals remained at baseline levels two weeks post-vaccination. Intracellular cytokine staining assay showed that the percentage of influenza virus–specific IFN-γ^+^CD4^+^ and IFN-γ^+^CD8^+^ T cells in lungs of SD/01/10-ca inoculated chickens were 1.14% and 0.87%, respectively, on day 7 after vaccination, which was significantly higher than the level before vaccination (0.16% positive for CD4^+^ T cells and 0.15% positive for CD8^+^ T cells). While in the mock-vaccinated group, the percentage of influenza virus–specific IFN-γ^+^CD4^+^ and IFN-γ^+^CD8^+^ T cells remained 0.17% positive for CD4^+^ T cells and 0.14% positive for CD8^+^ T cells on day 7 after vaccination (*P* < 0.05) (Fig. 2B). These results indicated that SD/01/10-ca was highly immunogenic and induced humoral and cellular immunity.

### Protective efficacy of H9N2 cold-adapted virus in chickens against homologous and heterogenous H9N2 viruses challenge

To determine the protective efficacy of SD/01/10-ca against wild type H9N2 viruses in chickens, homologous virus (SD/01/10-wt) and five heterogenous viruses belonging to different HA phylogenetic clades with different antigenicities (Fig. S2) circulating in China were used for the challenge study on day 14 after vaccination^15^,^18^.

Mock-vaccinated chickens infected with H9N2 viruses all exhibited anorexia, diarrhea, and increasing discharge in the oral cavity. In contrast, vaccinated animals inoculated with any of these viruses remained healthy during the observation period post-challenge. In the inoculated mock-vaccinated chickens, all six viruses were isolated from the oropharynx of all inoculated chickens on 3 and 5 days post challenge with mean titers ranging from 2.5 ± 0.4 to 6.3 ± 1.0 log_10_ EID_50_/mL, and five viruses maintained their growth in the oropharynx on day 7 post challenge (Table 3). All tested viruses could be detected from the cloacae of mock-vaccinated chickens. After placing mock-vaccinated chickens in contact with inoculated birds at 24 h post challenge, the five tested viruses maintained 100% transmission except A/chicken/Beijing/3/1999, which was only transmitted to three of the ten in-contact chickens (Table 3). In contrast, viruses were not isolated from the oropharynx or cloacae of inoculated vaccinated chickens, except for the A/chicken/Shandong/ZB/2007 inoculated group. Vaccination with SD/01/10-ca partially protected chickens from replication of A/chicken/Shandong/ZB/07 in the oropharynx and cloacae. Specifically, only two of the ten vaccinated birds shed viruses in the oropharynx and cloacae on day 3 post challenge, and titers were significantly lower than that of control group (*P* < 0.05). Viruses were not detected within vaccinated in-contact chickens in any groups. These results suggest that cold-adapted virus vaccination can protect chickens from homologous and heterologous H9N2 viruses infection and transmission.

### Humoral and cellular immune responses against challenged virus

**Figure 3:**
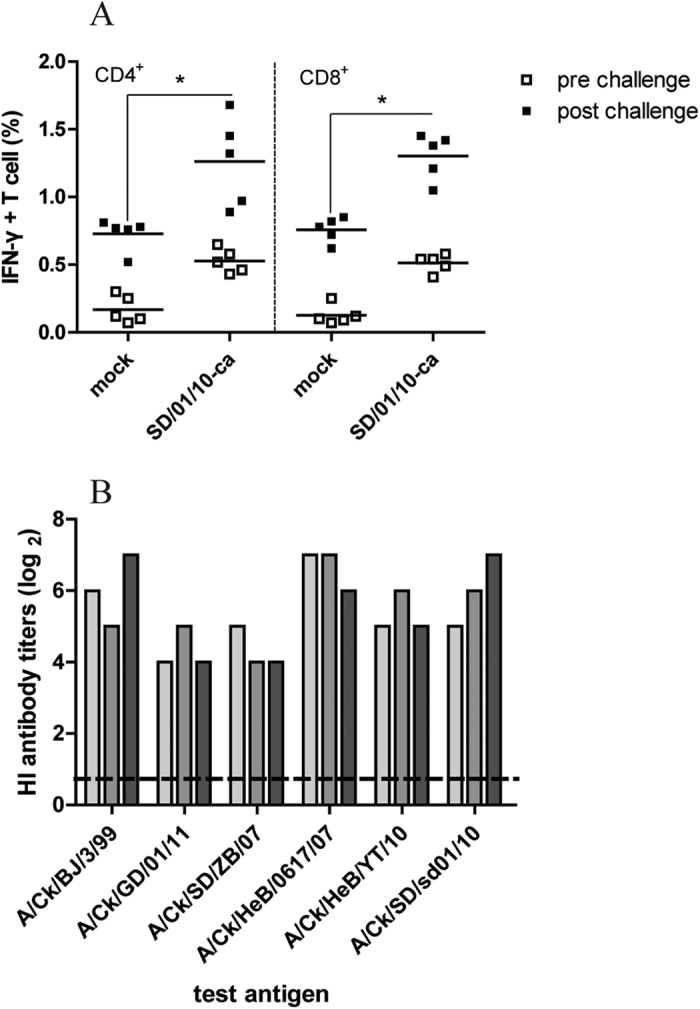
Immune responses after viruses challenge in chickens. (**A**) The frequency of IFN-γ^+^CD4^+^ or IFN-γ^+^CD8^+^ T cells in lungs. Chickens in groups of five were vaccinated with mock vaccine or SD/01/10-ca on day 0 and challenged intranasally with 10^6^ EID_50_ of A/chicken/Hebei/YT/2010 on day 14 after vaccination. Lungs were harvested on day 7 after challenge. IFN-γ^+^CD4^+^ or IFN-γ^+^CD8^+^ T cells in lungs stimulated with SD/01/10-wt were measured. Percentages of IFN-γ^+^CD4^+^ or IFN-γ^+^CD8^+^ T cells within CD4^+^ or CD8^+^ T cells were analyzed. *P < 0.05, by one-way analysis of variance of the difference between SD/01/10-ca and mock vaccine group on day 7 after challenge. (**B**) HI antibody responses to homologous and heterologous viruses after vaccination with SD/01/10-ca. Serum samples were collected on day 14 post immunization, and the reactivity of collected sera (n = 3) to SD/01/10-wt or five heterologous H9N2 influenza viruses was assessed by an HI assay. Abbreviations: BJ, Beijing; Ck, chicken; GD, Guangdong; HeB, Hebei; SD, Shandong.

To measure the cellular immune responses after virus challenge, lung cells of A/chicken/Hebei/YT/2010 challenged chickens harvested on day 7 after challenge were stimulated with A/chicken/Hebei/YT/2010 *in vivo* and IFN-γ-secreting T cells were identified by intracellular cytokine staining assay. The percentage of influenza virus–specific IFN-γ^+^CD4^+^ and IFN-γ^+^CD8^+^ T cells in the SD/01/10-ca vaccinated chickens (1.26% of all CD4^+^ T cells and 1.30% of all CD8^+^ T cells) was significantly higher (*P* < 0.05) than that in the mock groups (0.59% of all CD4^+^ T cells and 0.62% of all CD8^+^ T cells), and sharply increased relative to pre-challenge values (0.33% of all CD4^+^ T cells and 0.31% of all CD8^+^ T cells) (Fig. 3A), indicating a robust recall response. Furthermore, the cross-reactivity of HI antibody against homologous and five heterologous viruses was examined. Serum collected from chickens 2 weeks after vaccination inhibited hemagglutination by both homologues, with a mean titer of 2^6^, while they inhibited that of heterologous H9N2 influenza viruses with a mean titer ranged from 2^4.3^ to 2^6.7^ (Fig. 3B). Thus, the higher humoral and cellular immune responses might contribute to the effective protection conferred by SD/01/10-ca against homologous and heterologous H9N2 viruses.

## Discussion

In the present study, we generated a cold-adapted live attenuated H9N2 vaccine (SD/01/10-ca) by serial passages in embryonated eggs at successively lower temperatures, then evaluated its immunogenicity and efficacy. SD/01/10-ca possessed both the *ca* and *ts* phenotypes, and was attenuated in chickens. The vaccine induced humoral and T cell immune response in chickens after SD/01/10-ca immunization. Moreover, the vaccine completely protected chickens from the replication of five out of six tested viruses in the oropharynx and cloacae, and completely prevented the transmission of all tested viruses.

PB2, PB1 and NP have been shown to contribute to expression of the *ts* phenotype, and the PA gene specifies the *ca* phenotype of A/Ann Arbor/6/60 and A/Alaska/6/77 cold-adapted viruses^33^,^35^,^36^,^37^. Furthermore, amino acid 265S, 591I in PB2, 265N, 391E, 581G, 661T in PB1 and 34G in NP were found to contribute to the *ts* phenotype^33^,^38^, while amino acid 630R in PB2, 431M in PA, and 114A, 410H and 509T in NP were shown to be related to the *ca* phenotype of the commercial LAIV vaccine FluMist^39^. In our study, amino acid mutations were not only observed for PB2, PB1, PA and NP, but also for HA and NS1 proteins of the influenza SD/01/10 wild-type virus and its cold-adapted derivative. Moreover, all 13 mutations in cold-adapted H9N2 virus (SD/01/10-ca) were different from mutations in other cold-adapted viruses or identified mutations related to the *ts* and *ca* phenotype^29^,^35^,^40^. This might have been due to the different virus backgrounds and the different passage process used for cold adaptation. In fact, the viruses were passaged 30 times at 25 °C and we also sequenced the strains of passage 30 at 25 °C and found that the mutations were entirely consistent with the 13 mutations emerged in the ca strain at passage 25, indicating that the 13 mutations sequenced in the ca strain at passage 25 were stable mutations. The role of these mutations for *ts* and *ca* phenotypes needs to be further investigated.

When viruses were passaged 25 times at 25 °C in the present study, the viral titers in eggs ranged from 3.75 log_10_ EID_50_/mL to 6.25 log_10_ EID_50_/mL (data not shown). The introduction of acidic amino acids near the HA receptor binding site into the HA of 2009 pandemic H1N1 influenza virus and seasonal influenza viruses has been shown to improve vaccine virus growth in eggs or MDCK cells^41^. The mechanism was speculated to be that negatively charged residues in RBS decreased the HA and sialic acid interaction and thus facilitated the release of progeny viruses from infected cells for efficient multicycle replication^28^. The mutations HA-A150D and HA-L216F identified in SD/01/10-ca were also located in proximity to the RBS, and the HA-A150D changes resulted in the presence of the negatively charged acidic residue aspartate (D) on the surface of the HA. Therefore, these mutations in HA protein may be related to enhanced virus replication of SD/01/10-ca in eggs.

T cells elicited by conserved antigens shared between the vaccination and challenge viruses could be recruited to the site of infection to promote viral clearance and reduce disease severity^42^. The level of systemic and local T-cell responses was inversely correlated with the level of heterologous virus replication in the upper respiratory tract^43^. In this study, we showed that the exposure of chickens to heterologous virus challenge infection recalled the influenza virus–specific T-cell responses. The H9N2 cold-adapted vaccine reduced all challenging virus replication in the oropharynx and cloacae of vaccinated chickens. Thus, we speculated that T cell responses detected after virus challenge may play a key role in facilitating virus clearance in SD/01/10-ca vaccinated chickens. In addition, we showed that serum collected from SD/01/10-ca vaccinated chickens cross-reacted well with five heterologous H9N2 viruses. Therefore, protection against different H9N2 virus infection may be attributed to T-cells, as well as to cross-reactive binding antibodies.

The efficacy of a vaccine against different prevailing viruses, to a certain extent, determines whether it would be sufficiently effective to merit their widespread use in clinical applications. However, there have been relatively few evaluations of vaccine efficacy in the prevention of different circulating virus infection. Shin *et al*. showed that vaccination with inactivated H9N2 vaccine partially prevented replication of a H9N2 virus in the trachea of layers^44^. A recombinant *Lactobacillus plantarum* NC8 strain expressing the HA gene of H9N2 partially prevented replication of an H9N2 virus in the tracheas and lungs^45^. When cold adapted H9N2 vaccine vaccinated layers were challenged with one wild-type H9N2 influenza viruses, they were protected from the loss of egg production and virus replication in tracheas and cloacae^29^. In China, the prevailing H9N2 viruses belonged to different HA clades and exhibited distinct antigenic properties^15^,^18^. We evaluated the protective efficacy of SD/01/10-ca against H9N2 viruses belonging to different HA clades with different antigenicities. Our results suggest that vaccination with SD/01/10-ca completely protected chickens from the replication of all tested viruses, except A/chicken/Shandong/ZB/07, in the oropharynx and cloacae, and completely prevented the transmission of all tested viruses to the contact animals. These results indicated that SD/01/10-ca as a vaccine candidate elicited cross-reactive immune responses capable of protecting chickens against homologous and heterologous H9N2 avian influenza viruses.

In summary, the developed live cold-adapted attenuated H9N2 vaccine candidate that can confer protection of chickens from the challenge infection and transmission of different H9N2 viruses may have the potential to control outbreaks of H9N2 influenza viruses in China.

## Methods

### Ethics statement

All animal work was approved by the Beijing Association for Science and Technology (approval ID SYXK [Beijing] 2007-0023) and complied with the guidelines of Beijing laboratory animal welfare and ethics of Beijing Administration Committee of Laboratory Animals.

### Viruses and cells

H9N2 influenza virus, A/chicken/Shandong/sd01/2010 (SD/01/10-wt), was used for cold adaptation. Homologous virus SD/01/10-wt and five heterologous H9N2 influenza viruses belonging to different HA phylogenetic clades circulating in China, including A/chicken/Beijing/3/1999, A/chicken/Hebei/0617/2007, A/chicken/Shandong/ZB/2007, A/chicken/Hebei/YT/2010, and A/chicken/Guangdong/01/2011, were used for the challenge study^15^. All viruses were propagated in 10-day-old SPF embryonated chicken eggs. MDCK cells were grown in DMEM (Invitrogen, Carlsbad, CA, USA) containing 10% FBS (Invitrogen, Carlsbad, CA, USA).

### Cold adaptation of H9N2 influenza virus

Serial passages through ten-day-old SPF embryonated chicken eggs were performed by gradually reducing the incubating temperatures from 33 °C to 25 °C. Viruses were passaged 10 times at each temperature from 33 °C to 26 °C and 25 times at 25 °C^29^. For each passage, four eggs were inoculated with four hemagglutinin units (HAUs) (0.2 mL) of viruses diluted in PBS (phosphate-buffered saline, pH 7.4), then incubated for 48 h. After passage, viruses were further purified by limiting dilution in eggs at 25 °C 10 times^46^.

### Cold adaptation and temperature sensitivity phenotype analysis of the cold-adapted virus

The cold-adapted (*ca*) phenotype is defined as a less than 100-fold reduction of virus titer in eggs at 25 °C compared with that at 35 °C^36^. Viruses with the ability to form plaques in MDCK cells at 41 °C that were reduced 100-fold when compared with those at 33 °C and 39 °C and therefore considered temperature sensitive (*ts*)^36^. The *ca* and *ts* phenotype were tested as previously described^29^.

### Genetic analysis of cold-adapted H9N2 influenza virus

Viral RNA was extracted using a QIAamp Viral RNA Minikit (Qiagen, Hilden, Germany), and standard RT-PCR was performed with primers specific for H9N2 influenza virus using the One Step RT-PCR Kit (Qiagen, Hilden, Germany). Full-genome DNA samples were Sanger sequenced, after which sequence alignment was conducted using ClustalW (http://www.ebi.ac.uk/clustalw).

### Replication and transmission of cold-adapted H9N2 influenza virus in chickens

Three-week-old SPF white Leghorn chickens (Beijing Merial Vital Laboratory Animal Technology Co. Ltd., Beijing, China) were intranasally inoculated with 1×10^6^ EID_50_ of SD/01/10-ca or SD/01/10-wt. Tracheas and lungs were collected on 2, 3, 5 and 7 days post inoculation, respectively. For the transmission experiment, six naive in-contact chickens were placed in physical contact with each inoculated group at 24 h post inoculation. Oropharyngeal and cloacal swabs were then collected on 2 and 4 days post inoculation. Collected organs and swabs were titered by EID_50_ assay^47^.

### Hemagglutination-inhibition assay

The hemagglutination inhibition (HI) antibody titers of the sera were determined using 1% chicken red blood cells as previously described^48^.

### Intracellular staining of T lymphocytes expressing IFN-γ

Lung tissue was digested with 50 U/mL DNase (Sigma-Aldrich, St. Louis, MO, USA) and 1 mg/mL collagenase A (Sigma Aldrich, St. Louis, MO, USA) in RPMI 1640 medium (Invitrogen, Carlsbad, CA, USA) containing 5% FBS (Invitrogen, Carlsbad, CA, USA) for 1 h at 37 °C. Digested lungs were homogenized and red blood cells were lysed. The cell suspensions from the lungs were then incubated in RPMI 1640 cell-culture medium (10% FBS, 1% Penicillin-Streptomycin) for 6 h at 37 °C under 5% CO_2_ in the presence of SD/01/10-wt or A/chicken/Hebei/YT/2010 at a multiplicity of infection of 1, 10 U/mL rhIL-2 (PeProTech, Rocky Hill, NJ, USA), and 1 μL/mL Brefeldin A (eBioscience, San Diego, CA, USA) at 0 h for CD8^+^ responses, or 2 h for CD4^+^ responses. At 6 h, cells were washed with PBS containing 2% FBS, then stained with anti-CD8-SPRD and anti-CD4-PE (Southern Biotech, Birmingham, AL, USA) for 30 min on ice. For intracellular IFN-γ staining, cells were permeabilized using Cytofix/Cyoperm (BD Biosciences, San Diego, CA, USA), followed by staining with rabbit anti-chicken IFN-γ antibody (AbD Serotec, Raleigh, NC, USA) for 30 min on ice, then stained with FITC-labeled anti-rabbit antibody (Abcam, Cambridge, UK) for 30 min on ice. The stained cells were evaluated by flow cytometry (BD Biosciences, San Diego, CA, USA) for expression of CD4, CD8, and IFN-γ, after which the data were analyzed using the FlowJo Software (Tree Star, San Carlos, CA, USA).

### Protective efficacy of the cold-adapted H9N2 influenza virus in chickens

Groups of 3-week-old SPF white Leghorn chickens were vaccinated intranasally with 10^6^ EID_50_ of SD/01/10-ca or PBS (mock immunized) in 0.2 mL. In inoculated groups, the chickens were challenged intranasally with 10^6^ EID_50_ of the homologous virus (SD/01/10-wt) or heterologous viruses, A/chicken/Beijing/3/1999, A/chicken/Hebei/0617/2007, A/chicken/Shandong/ZB/2007, A/chicken/Hebei/YT/2010, and A/chicken/Guangdong/01/2011, on day 14 post vaccination. In contact groups, chickens were placed in physical contact with inoculated birds at 24 h post challenge. Oropharyngeal and cloacal swabs of all inoculated and contact chickens were collected on 3, 5, and 7 days post challenge for viral detection and titration.

### Statistical analyses

Statistically significant differences between experimental groups were determined using the analysis of variance (ANOVA) method with the GraphPad Prism software (GraphPad Software Inc., La Jolla, CA, USA). Differences were considered statistically significant at *P* < 0.05.

## Additional Information

**How to cite this article**: Wei, Y. *et al*. Generation and protective efficacy of a cold-adapted attenuated avian H9N2 influenza vaccine. *Sci. Rep.* **6**, 30382; doi: 10.1038/srep30382 (2016).

## Supplementary Material

Supplementary Figure S1

Supplementary Figure S2

## Figures and Tables

**Table 1:** Amino acid differences between wild-type and cold-adapted H9N2 influenza viruses. ^a^H9 numbering (H3 numbering).

Protein	Amino acid position	SD/01/10-wt	SD/01/10-ca
PB2	136	R	Q
	290	G	C
	505	R	L
	654	P	S
PB1	292	N	K
	361	S	N
	739	E	K
PA	27	D	N
	319	E	K
HA	150 (160) ^a^	A	D
	216 (226) ^a^	L	F
NP	17	G	R
NS1	184	G	R

**Table 2:** Replication and transmission of cold-adapted H9N2 virus in chickens.

Virus	Group	Virus shedding in oropharynx on day^a^		Virus shedding in cloaca on day^a^		Virus replication in tracheas on day^a^				Virus replication in lungs on day^a^			
		2	4	2	4	2	3	5	7	2	3	5	7
SD/01/10-wt	Inoculation	6/6 (5.3 ± 0.6)	6/6 (4.9 ± 0.5)	6/6 (3.3 ± 0.7)	4/6 (2.2 ± 0.3)	6/6 (3.4 ± 0.4)^b^	6/6 (4.4 ± 0.4)	6/6 (3.6 ± 0.2)	1/6 (1.5)	6/6 (4.2 ± 0.4)	6/6 (5.3 ± 0.9)	6/6 (3.5 ± 0.3)	1/6 (1.8)
	Contact	6/6 (4.8 ± 0.3)	6/6 (5.2 ± 0.4)	6/6 (3.7 ± 0.2)	2/6 (2.0 ± 0.4)	/	/	/	/	/	/	/	/
SD/01/10-ca	Inoculation	5/6 (1.3 ± 0.5)	0/6	0/6	0/6	6/6 (1.8 ± 0.4)	0/6	0/6	0/6	0/6	0/6	0/6	0/6
	Contact	0/6	0/6	0/6	0/6	/	/	/	/	/	/	/	/

^a^3-week-old SPF chickens were inoculated intranasally with 10^6^ EID_50_ in 0.2 mL of SD/01/10-ca or SD/01/10-wt. At 24 h post inoculation, six naive in-contact chickens were added to each infected group. Six inoculated chickens in each group were killed on day 2, 3, 5, 7 post inoculation and their organs were collected. Oropharyngeal and cloacal swabs of directly infected chickens and in-contact chickens were collected on 2 and 4 days post inoculation. Collected organs and swabs were titrated in eggs.

^b^No. of chickens positive for virus/total no. of chickens tested (mean titers [log_10_ EID_50_/mL]  ± standard errors).

**Table 3:** Vaccine protection against different prevailing H9N2 influenza viruses.

Virus	Vaccination	Group	Virus shedding in oropharynx (days post challenge)^a^			Virus shedding in cloaca (days post challenge)^a^		
			3	5	7	3	5	7
A/chicken/Beijing/3/1999	**−**	Inoculated	10/10 (3.5 ± 0.7)^b^	10/10 (2.5 ± 0.4)	0/10	8/10 (1.8 ± 0.3)	0/10	0/10
		Contact	5/10(1.8 ± 0.4)	0/10	0/10	0/10	0/10	0/10
	**+**	Inoculated	0/10	0/10	0/10	0/10	0/10	0/10
		Contact	0/10	0/10	0/10	0/10	0/10	0/10
A/chicken/Hebei/0617/2007	**−**	Inoculated	10/10 (4.5 ± 0.7)	10/10 (3.8 ± 1.0)	1/10 (1.8)	7/10 (1.2 ± 0.5)	3/10 (1.8 ± 0.2)	0/10
		Contact	10/10 (4.8 ± 0.9)	10/10 (5.1 ± 0.8)	10/10 (3.8 ± 0.4)	7/10 (1.5 ± 0.4)	0/10	0/10
	**+**	Inoculated	0/10	0/10	0/10	0/10	0/10	0/10
		Contact	0/10	0/10	0/10	0/10	0/10	0/10
A/chicken/Shandong/ZB/2007	**−**	Inoculated	10/10 (4.5 ± 0.6)	10/10 (3.8 ± 0.8)	5/10 (1.8 ± 0.5)	7/10 (1.3 ± 0.7)	3/10 (1.6 ± 0.6)	0/10
		Contact	10/10 (4.1 ± 0.8)	10/10 (4.5 ± 0.6)	10/10 (2.4 ± 0.8)	6/10 (1.2 ± 0.7)	4/10 (1.8 ± 0.7)	0/10
	**+**	Inoculated	3/10(1.5 ± 0.2)	0/10	0/10	4/10(1.5 ± 0.4)	0/10	0/10
		Contact	0/10	0/10	0/10	0/10	0/10	0/10
A/chicken/Hebei/YT/2010	**−**	Inoculated	10/10 (6.3 ± 1.0)	10/10 (4.8 ± 0.8)	6/10 (2.8 ± 1.0)	10/10 (3.7 ± 0.8)	10/10 (3.5 ± 0.7)	6/10 (1.5 ± 0.8)
		Contact	10/10 (5.7 ± 0.8)	10/10 (5.3 ± 0.7)	8/10 (3.5 ± 0.7)	10/10 (2.8 ± 0.5)	10/10 (1.8 ± 0.9)	7/10 (2.5 ± 0.6)
	**+**	Inoculated	0/10	0/10	0/10	0/10	0/10	0/10
		Contact	0/10	0/10	0/10	0/10	0/10	0/10
A/chicken/Guangdong/01/2011	**−**	Inoculated	10/10 (5.8 ± 0.7)	10/10 (3.8 ± 0.6)	4/10 (1.8 ± 0.7)	10/10 (2.5 ± 0.7)	6/10 (2.8 ± 0.5)	0/10
		Contact	10/10 (3.8 ± 0.8)	10/10 (4.5 ± 1.0)	3/10 (4.5 ± 0.8)	10/10 (1.8 ± 0.6)	3/10 (1.5 ± 0.3)	0/10
	**+**	Inoculated	0/10	0/10	0/10	0/10	0/10	0/10
		Contact	0/10	0/10	0/10	0/10	0/10	0/10
A/chicken/Shandong/sd01/2010	**−**	Inoculated	10/10 (5.7 ± 0.8)	10/10 (3.5 ± 0.9)	3/10 (1.5 ± 0.4)	10/10 (2.7 ± 0.9)	4/10 (2.5 ± 0.6)	3/10 (1.8 ± 0.4)
		Contact	10/10 (5.3 ± 0.5)	10/10 (4.3 ± 0.7)	6/10 (2.5 ± 0.6)	10/10 (2.8 ± 0.7)	7/10 (1.8 ± 0.9)	0/10
	**+**	Inoculated	0/10	0/10	0/10	0/10	0/10	0/10
		Contact	0/10	0/10	0/10	0/10	0/10	0/10

^a^Groups of 3-week-old SPF white Leghorn chickens were immunized intranasally with 0.2 mL 10^6^ EID_50_ of cold-adapted H9N2 viruses or phosphate-buffered saline (mock immunized). Chickens were challenged intranasally with 10^6^ EID_50_ of the homologous or heterologous viruses on day 14 post immunization. At 24 h after challenge, in-contact chickens were placed in physical contact with challenged birds. Virus titers in oropharyngeal and cloacal swabs on 3, 5, and 7 days post challenge were determined.

^b^No. of chickens positive for virus/total no. of chickens tested (mean titers [log_10_ EID_50_/mL] ± standard errors).
