# Supplementary material for: Generation and protective efficacy of a cold-adapted attenuated avian H9N2 influenza vaccine
Source: Sci Rep. 2016 Jul 26;6:30382. doi: 10.1038/srep30382 (PMC4960571; doi:10.1038/srep30382)
Supplement: Supplementary Figure S1 [file srep30382-s1.pdf]

Supplementary Figure 1

for

Generation and protective efficacy of a cold-adapted attenuated avian H9N2 influenza vaccine

Yandi Wei, Lu Qi, Huijie Gao, Honglei Sun, Juan Pu, Yipeng Sun, and Jinhua Liu

A

```
       1        10        20        30        40        50        60
WTPB2  MERIKELRDLMSQSRTREILTKTTVDHMAIIKKYTSGRQEKNPALRMKWMMAMKYPITAN
CAPB2  MERIKELRDLMSQSRTREILTKTTVDHMAIIKKYTSGRQEKNPALRMKWMMAMKYPITAN

                70        80        90       100       110       120
WTPB2  KRIMEMIPERNEQGQTLWSKTNDAGSDRVMVSPLAVTWWNRNGPTTSTVHYPKVYKTYFE
CAPB2  KRIMEMIPERNEQGQTLWSKTNDAGSDRVMVSPLAVTWWNRNGPTTSTVHYPKVYKTYFE

               130       140       150       160       170       180
WTPB2  KVERLKHGTFGPVHFRNQVKIRRRVDINPGHADLSAKEAQDVIMEVVFPNEVGARILTSE
CAPB2  KVERLKHGTFGPVHFQNQVKIRRRVDINPGHADLSAKEAQDVIMEVVFPNEVGARILTSE

               190       200       210       220       230       240
WTPB2  SQLTITKEKKEELQDCKIAPLMVAYMLERELVRKTRFLPVAGGTSSVYIEVLHLTQGTCW
CAPB2  SQLTITKEKKEELQDCKIAPLMVAYMLERELVRKTRFLPVAGGTSSVYIEVLHLTQGTCW

               250       260       270       280       290       300
WTPB2  EQMYTPGGKVRNDDVDQSLIIAARNIVRRAIVSADPLASLLEMCHSTQIGGVRMVDILRQ
CAPB2  EQMYTPGGKVRNDDVDQSLIIAARNIVRRAIVSADPLASLLEMCHSTQICGVRMVDILRQ

               310       320       330       340       350       360
WTPB2  NPTEEQAVDICKAAMGLRISSSFSFGGFTFKRTSGSSVKREEEVLTGNLQTLKIRVHEGY
CAPB2  NPTEEQAVDICKAAMGLRISSSFSFGGFTFKRTSGSSVKREEEVLTGNLQTLKIRVHEGY

               370       380       390       400       410       420
WTPB2  EEFTMVGRRATAILRKATRRLIQLIVSGKDEQSIAEAIIVAMVFSQEDCMIKAVRGDLNF
CAPB2  EEFTMVGRRATAILRKATRRLIQLIVSGKDEQSIAEAIIVAMVFSQEDCMIKAVRGDLNF

               430       440       450       460       470       480
WTPB2  VNRANQRLNPMHQLLRHFQKDAKVLFQNWGIEPIDNVMGMIGILPDMTPSTEMSLRGVRV
CAPB2  VNRANQRLNPMHQLLRHFQKDAKVLFQNWGIEPIDNVMGMIGILPDMTPSTEMSLRGVRV

               490       500       510       520       530       540
WTPB2  SKMGVDEYSSAERVVVSIDRFLRVRDQRGNVLLSPEEVSETQGTEKLTITYSSSMMWEIN
CAPB2  SKMGVDEYSSAERVVVSIDRFLRVLDQRGNVLLSPEEVSETQGTEKLTITYSSSMMWEIN

               550       560       570       580       590       600
WTPB2  GPESVLVNTYQWIIRNWETVKIQWSQDPTMLYNKMEFEPFQSLVPKAARGQYSGFVRVLF
CAPB2  GPESVLVNTYQWIIRNWETVKIQWSQDPTMLYNKMEFEPFQSLVPKAARGQYSGFVRVLF

               610       620       630       640       650       660
WTPB2  QQMRDVLGTFDTVQIIKLLPFAAAPPEQSRMQFSSLTVNVRGSGMRIVVRGNSPVFNYNK
CAPB2  QQMRDVLGTFDTVQIIKLLPFAAAPPEQSRMQFSSLTVNVRGSGMRIVVRGNSSVFNYNK

               670       680       690       700       710       720
WTPB2  ATKRLTVLGKDAGALMEDPDEGTSGVESAVLRGFLILGKEDKRYGPALSINELSNLAKGE
CAPB2  ATKRLTVLGKDAGALMEDPDEGTSGVESAVLRGFLILGKEDKRYGPALSINELSNLAKGE

               730       740       750
WTPB2  KANVLIGQGDVVLVMKRKRDSSILTDSQTATKRIRMAIN
CAPB2  KANVLIGQGDVVLVMKRKRDSSILTDSQTATKRIRMAIN
```

B

```
       1        10        20        30        40        50        60
WTPB1  MDVNPTLLFLKVPVQNAISTTFPYTGDPPYSHGTGTGYTMDTVNRTHKYSEKGKWTTNTE
CAPB1  MDVNPTLLFLKVPVQNAISTTFPYTGDPPYSHGTGTGYTMDTVNRTHKYSEKGKWTTNTE

                70        80        90       100       110       120
WTPB1  TGAPQLNPIDGPLPEDNEPSGYAQTDCVLEAMAFLEESHPGIFENSCLETMEIVQQTRVD
CAPB1  TGAPQLNPIDGPLPEDNEPSGYAQTDCVLEAMAFLEESHPGIFENSCLETMEIVQQTRVD

               130       140       150       160       170       180
WTPB1  KLTQGRQTYDWTLNRNQPAATALANTIEVFRSNGLTANESGRLIDFLKDVMDSMDKEEME
CAPB1  KLTQGRQTYDWTLNRNQPAATALANTIEVFRSNGLTANESGRLIDFLKDVMDSMDKEEME

               190       200       210       220       230       240
WTPB1  ITTHFQRKRRVRDNMTKKMVTQRTIGKKKQRLNKRSYLIRALTLNTMTKDAERGKLKRRA
CAPB1  ITTHFQRKRRVRDNMTKKMVTQRTIGKKKQRLNKRSYLIRALTLNTMTKDAERGKLKRRA

               250       260       270       280       290       300
WTPB1  IATPGMQIRGFVYFVEALARSICEKLEQSGLPVGGNEKKAKLANVVRKMMTNSQDTELSF
CAPB1  IATPGMQIRGFVYFVEALARSICEKLEQSGLPVGGNEKKAKLANVVRKMMTKSQDTELSF

               310       320       330       340       350       360
WTPB1  TITGDNTKWNENQNPRMFLAMITYITRNQPEWFRNVLSIAPIMFSNKMARLGKGYMFESK
CAPB1  TITGDNTKWNENQNPRMFLAMITYITRNQPEWFRNVLSIAPIMFSNKMARLGKGYMFESK

               370       380       390       400       410       420
WTPB1  SMKLRTQVPAEMLANIDLKYFNKSTREKIEKIRPLLIDGTASLSPGMMMGMFNMLSTVLG
CAPB1  NMKLRTQVPAEMLANIDLKYFNKSTREKIEKIRPLLIDGTASLSPGMMMGMFNMLSTVLG

               430       440       450       460       470       480
WTPB1  VSILNLGQKKYTKNTYWWDGLQSSDDFALIVNAPNHEGIQAGVDRFYRTCKLVGINMSKK
CAPB1  VSILNLGQKKYTKNTYWWDGLQSSDDFALIVNAPNHEGIQAGVDRFYRTCKLVGINMSKK

               490       500       510       520       530       540
WTPB1  KSYINRTGTFEFTSFFYRYGFVANFSMELPSFGVSGINESADMSIGVTVIKNNMINNDLG
CAPB1  KSYINRTGTFEFTSFFYRYGFVANFSMELPSFGVSGINESADMSIGVTVIKNNMINNDLG

               550       560       570       580       590       600
WTPB1  PATAQMALQLFIKDYRYTYRCHRGDTQIQTRRAFELKKLWEQTRSKAGLLVSDGGPNLYN
CAPB1  PATAQMALQLFIKDYRYTYRCHRGDTQIQTRRAFELKKLWEQTRSKAGLLVSDGGPNLYN

               610       620       630       640       650       660
WTPB1  IRNLHIPEVCLKWELMDEDYQGRLCNPINPFVSHKEIDSVNNAVVMPAHGPAKSMEYDAV
CAPB1  IRNLHIPEVCLKWELMDEDYQGRLCNPINPFVSHKEIDSVNNAVVMPAHGPAKSMEYDAV

               670       680       690       700       710       720
WTPB1  ATTHSWIPKRNRSILNTSQRGVLEDEQMYQKCCNLFEKFFPSSSYRRPVGISSMVEAMVS
CAPB1  ATTHSWIPKRNRSILNTSQRGVLEDEQMYQKCCNLFEKFFPSSSYRRPVGISSMVEAMVS
```

```
                   730       740       750
                     .         .         .
WTPB1    RARIDARIDFESGGIKKEEFAEIMKICSTIEELRRQK
CAPB1    RARIDARIDFESGGIKKEKFAEIMKICSTIEELRRQK
```

C

```
       1        10        20        30        40        50        60
WTPA   MEDFVRQCFNPMIVELAEKAMKEYGEDPKIETNKFASICTHLEVCFMYSDFHFIDERGES
CAPA   MEDFVRQCFNPMIVELAEKAMKEYGENPKIETNKFASICTHLEVCFMYSDFHFIDERGES

                70        80        90       100       110       120
WTPA   TIIESGDPNALLKHRFEIIEGRDRTMAWTVVNSICNTTGVEKPKFLPDLYDYKENRFIEI
CAPA   TIIESGDPNALLKHRFEIIEGRDRTMAWTVVNSICNTTGVEKPKFLPDLYDYKENRFIEI

               130       140       150       160       170       180
WTPA   GVTRREVHIYYLEKANKIKSEKTHIHIFSFTGEEMATKADYTLDEESRARIKTRLFTIRH
CAPA   GVTRREVHIYYLEKANKIKSEKTHIHIFSFTGEEMATKADYTLDEESRARIKTRLFTIRH

               190       200       210       220       230       240
WTPA   EMASRGLWDSFRQSERGEETIEERFEITGTMRRLADQSLPPNFSSLENFRAYVDGFEPNG
CAPA   EMASRGLWDSFRQSERGEETIEERFEITGTMRRLADQSLPPNFSSLENFRAYVDGFEPNG

               250       260       270       280       290       300
WTPA   CIEGKLSQMSKEVNARIEPFLRTTPRPLRLPNGPPCSQRSKFLLMDALKLSIEDPSHEGE
CAPA   CIEGKLSQMSKEVNARIEPFLRTTPRPLRLPNGPPCSQRSKFLLMDALKLSIEDPSHEGE

               310       320       330       340       350       360
WTPA   GIPLYDAIKCMKTFFGWKEPNIIKPHEKGINPNYLLTWKQVLAELQDIENEEKIPKTKNM
CAPA   GIPLYDAIKCMKTFFGWKKPNIIKPHEKGINPNYLLTWKQVLAELQDIENEEKIPKTKNM

               370       380       390       400       410       420
WTPA   KKTSQLKWALGENMAPEKVDFEDCKDVNDLKQYDSDEPEPRSLACWIQNEFNKACELTDS
CAPA   KKTSQLKWALGENMAPEKVDFEDCKDVNDLKQYDSDEPEPRSLACWIQNEFNKACELTDS

               430       440       450       460       470       480
WTPA   SWVELDEIGEDVAPIEHIASMRRNYFTAEVSHCRATEYIMKGVYINTALLNASCAAMDDF
CAPA   SWVELDEIGEDVAPIEHIASMRRNYFTAEVSHCRATEYIMKGVYINTALLNASCAAMDDF

               490       500       510       520       530       540
WTPA   QLIPMISKCRTKEGRRKTNLYGFIIKGRSHLRNDTDVVNFVSMEFSLTDPRLEPHKWEKY
CAPA   QLIPMISKCRTKEGRRKTNLYGFIIKGRSHLRNDTDVVNFVSMEFSLTDPRLEPHKWEKY

               550       560       570       580       590       600
WTPA   CVLEIGDMLLRTAVGQVSRPMFLYVRTNGTSKIKMKWGMEMRRCLLQSLQQIESMIEAES
CAPA   CVLEIGDMLLRTAVGQVSRPMFLYVRTNGTSKIKMKWGMEMRRCLLQSLQQIESMIEAES

               610       620       630       640       650       660
WTPA   SVKEKDLTKEFFENKSETWPIGESPKGVEEGSIGKVCRTLLAKSVFNSLYASPQLEGFSA
CAPA   SVKEKDLTKEFFENKSETWPIGESPKGVEEGSIGKVCRTLLAKSVFNSLYASPQLEGFSA

               670       680       690       700       710
WTPA   ESRKLLLIVQALRDNLEPGTFDLEGLYEAIEECLINDPWVLLNASWFNSFLTHAQR
CAPA   ESRKLLLIVQALRDNLEPGTFDLEGLYEAIEECLINDPWVLLNASWFNSFLTHAQR
```

D

```
        1        10        20        30        40        50        60
WTHA    DKICIGYQSTNSTETVDTLTENNVPVTHAKELLHTEHNGMLCATSLGHPLILDTCTIEGL
CAHA    DKICIGYQSTNSTETVDTLTENNVPVTHAKELLHTEHNGMLCATSLGHPLILDTCTIEGL

                 70        80        90       100       110       120
WTHA    IYGNPSCDLLLGGREWSYIVERPSAVNGLCYPGNVENLEELRSLFSSARSYQRIQIFPDT
CAHA    IYGNPSCDLLLGGREWSYIVERPSAVNGLCYPGNVENLEELRSLFSSARSYQRIQIFPDT

                130       140       150       160       170       180
WTHA    IWNVSYSGTSKACSDSFYRSMRWLTQKNNAYPIQDAQYTNNQEKNILFMWGINHPPTDTA
CAHA    IWNVSYSGTSKACSDSFYRSMRWLTQKNNDYPIQDAQYTNNQEKNILFMWGINHPPTDTA

                190       200       210       220       230       240
WTHA    QTNLYTRTDTTTSVATEEINRTFKPLIGPRPLVNGLQGRIDYYWSVLKPGQTLRIRSNGN
CAHA    QTNLYTRTDTTTSVATEEINRTFKPLIGPRPLVNGFQGRIDYYWSVLKPGQTLRIRSNGN

                250       260       270       280       290       300
WTHA    LIAPWYGHILSGESHGRILKTDLKRGSCTVQCQTEKGGLNTTLPFQNVSKYAFGNCSKYI
CAHA    LIAPWYGHILSGESHGRILKTDLKRGSCTVQCQTEKGGLNTTLPFQNVSKYAFGNCSKYI

                310       320       330       340       350       360
WTHA    GIKSLKLAVGLRNVPSRSSRGLFGAIAGFIEGGWSGLVAGWYGFQHSNDQGVGMAADRDS
CAHA    GIKSLKLAVGLRNVPSRSSRGLFGAIAGFIEGGWSGLVAGWYGFQHSNDQGVGMAADRDS

                370       380       390       400       410       420
WTHA    TQKAIDKITSKVNNIVDKMNKQYEIIDHEFSEVETRLNMINNKIDDQIQDIWAYNAELLV
CAHA    TQKAIDKITSKVNNIVDKMNKQYEIIDHEFSEVETRLNMINNKIDDQIQDIWAYNAELLV

                430       440       450       460       470       480
WTHA    LLENQKTLDEHDANVNNLYNKVKRALGSNAVEDGKGCFELYHKCDDQCMETIRNGTYNRR
CAHA    LLENQKTLDEHDANVNNLYNKVKRALGSNAVEDGKGCFELYHKCDDQCMETIRNGTYNRR

                490       500       510       520       530       540
WTHA    KYQEESKLERQKIEGVKLESEGTYKILTIYSTVASSIVIAMGFAAFLFWAMSNGSCRCNI
CAHA    KYQEESKLERQKIEGVKLESEGTYKILTIYSTVASSIVIAMGFAAFLFWAMSNGSCRCNI

WTHA    CI
CAHA    CI
```

E

```
      1        10        20        30        40        50        60
WTNP  MASQGTKRSYEQMETGGDRQNATEIRASVGRMVSGIGRFYIQMCTELKLSDNEGRLIQNS
CANP  MASQGTKRSYEQMETGRDRQNATEIRASVGRMVSGIGRFYIQMCTELKLSDNEGRLIQNS

               70        80        90       100       110       120
WTNP  ITIERMVLSAFDERRNRYLEEHPSAGKDPKKTGGPIYRRRDGKWVRELILYDKEEIRRIW
CANP  ITIERMVLSAFDERRNRYLEEHPSAGKDPKKTGGPIYRRRDGKWVRELILYDKEEIRRIW

              130       140       150       160       170       180
WTNP  RQANNGEDATAGLTHLMIWHSNLNDATYQRTRALVRTGMDPRMCSLMQGSTLPRRSGAAG
CANP  RQANNGEDATAGLTHLMIWHSNLNDATYQRTRALVRTGMDPRMCSLMQGSTLPRRSGAAG

              190       200       210       220       230       240
WTNP  AAVKGIGTMVMELIRMIKRGINDRNFWRGENGRRTRIAYERMCNILKGKFQTAAQRAMMD
CANP  AAVKGIGTMVMELIRMIKRGINDRNFWRGENGRRTRIAYERMCNILKGKFQTAAQRAMMD

              250       260       270       280       290       300
WTNP  QVRESRNPGNAEIEDLIFLARSALILRGSVAHKSCLPACVYGLAVASGYDFEREGYSLVG
CANP  QVRESRNPGNAEIEDLIFLARSALILRGSVAHKSCLPACVYGLAVASGYDFEREGYSLVG

              310       320       330       340       350       360
WTNP  IDPFRLLQNSQVFSLIRPNENPAHKSQLVWMACHSAAFEDLRVSSFIRGTRMVPRGQLST
CANP  IDPFRLLQNSQVFSLIRPNENPAHKSQLVWMACHSAAFEDLRVSSFIRGTRMVPRGQLST

              370       380       390       400       410       420
WTNP  RGVQIASNENIEAMDSNTLELRSRYWAIRTKSGGNTNQQRASAGQVSVQPTFSVQRNLPF
CANP  RGVQIASNENIEAMDSNTLELRSRYWAIRTKSGGNTNQQRASAGQVSVQPTFSVQRNLPF

              430       440       450       460       470       480
WTNP  ERATIMAAFTGNTEGRTSDMRTEIIRMMESARPEDVSFQGRGVFELSDEKATNPIVPSFD
CANP  ERATIMAAFTGNTEGRTSDMRTEIIRMMESARPEDVSFQGRGVFELSDEKATNPIVPSFD

              490
WTNP  MNNEGSYFFGDNAEEYDN
CANP  MNNEGSYFFGDNAEEYDN
```

F

```
         1        10        20        30        40        50        60
WTNS1    MDSNTVSSFQVDCFLWHVRKRFADQELGDAPFLDRLRRDQKSLRGRSSTLGLDIRTATRE
CANS1    MDSNTVSSFQVDCFLWHVRKRFADQELGDAPFLDRLRRDQKSLRGRSSTLGLDIRTATRE

                  70        80        90       100       110       120
WTNS1    GKHIVERILEEESDEAFKMTIASVPAPRYLTDMTLEEMSRDWLMLIPKQKVTGSLCIRME
CANS1    GKHIVERILEEESDEAFKMTIASVPAPRYLTDMTLEEMSRDWLMLIPKQKVTGSLCIRME

                 130       140       150       160       170       180
WTNS1    QAIVDKNITLKANFIVIFNRLEALILLRAFTDEGAIVGEISPLPSLPGHTDKDVKNAIGV
CANS1    QAIVDKNITLKANFIVIFNRLEALILLRAFTDEGAIVGEISPLPSLPGHTDKDVKNAIGV

                 190       200       210
WTNS1    LIGGFEWNDNTVRVSETLQRFAWRSSDEDGRPPLSPK
CANS1    LIGRFEWNDNTVRVSETLQRFAWRSSDEDGRPPLSPK
```

Fig. S1. Amino acid sequences of the SD/01/10- wt and ca mutant viruses.

Identical sequences of PB2 (A), PB1 (B), PA (C), HA (D), NP (E), NS1(F) are shown in red, amino acid changes detected in the ca mutant viruses are shown in white.
