# Supplementary material for: Generation and protective efficacy of a cold-adapted attenuated avian H9N2 influenza vaccine
Source: Sci Rep. 2016 Jul 26;6:30382. doi: 10.1038/srep30382 (PMC4960571; doi:10.1038/srep30382)
Supplement: Supplementary Figure S2 [file srep30382-s2.pdf]

Supplementary Figure 2

for

Generation and protective efficacy of a cold-adapted attenuated avian H9N2 influenza vaccine

Yandi Wei, Lu Qi, Huijie Gao, Honglei Sun, Juan Pu, Yipeng Sun, and Jinhua Liu

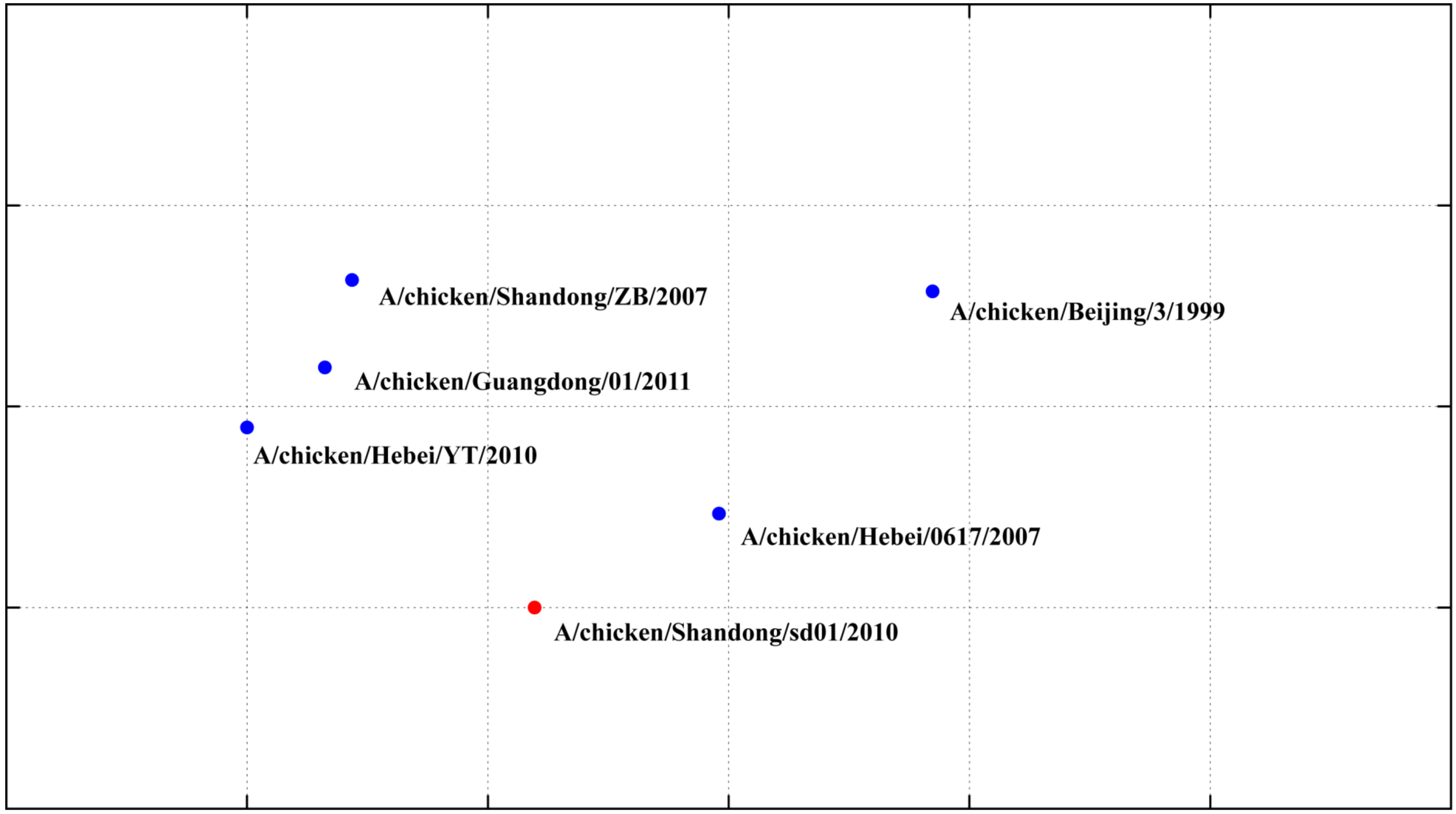

Fig. S2. Antigenic cartography representation of viruses used in challenge experiment. The map was produced using AntigenMap

(http://sysbio.cvm.msstate.edu/AntigenMap). One unit (grid) represents a 2-fold change in the HI assay results. Red circle indicate the vaccine strain.
